# Co-infection restrains *Litomosoides sigmodontis* filarial load and plasmodial *P. yoelii* but not *P. chabaudi* parasitaemia in mice

**DOI:** 10.1051/parasite/2014017

**Published:** 2014-04-10

**Authors:** Gregory Karadjian, Dominique Berrebi, Nathalie Dogna, Nathaly Vallarino-Lhermitte, Odile Bain, Irène Landau, Coralie Martin

**Affiliations:** 1 UMR 7245 MCAM MNHN CNRS, Muséum National d’Histoire Naturelle 61 rue Buffon, CP 52 75231 Paris Cedex 05 France; 2 Service d’Anatomie et de Cytologie Pathologique, Paris, Hôpital Robert Debré, Assistance Publique-Hôpitaux de Paris France, and EA3102, Université Paris 7 France

**Keywords:** Coinfection, Murine, *Litomosoides sigmodontis*, Plasmodium, Parasitology, Anatomopathology

## Abstract

Infection with multiple parasite species is clearly the norm rather than the exception, in animals as well as in humans. Filarial nematodes and *Plasmodium* spp. are important parasites in human public health and they are often co-endemic. Interactions between these parasites are complex. The mechanisms underlying the modulation of both the course of malaria and the outcome of filarial infection are poorly understood. Despite increasing activity in recent years, studies comparing co- and mono-infections are very much in their infancy and results are contradictory at first sight. In this study we performed controlled and simultaneous co-infections of BALB/c mice with *Litomosoides sigmodontis* filaria and with *Plasmodium* spp. (*Plasmodium yoelii* 17 XNL or *Plasmodium chabaudi* 864VD). An analysis of pathological lesions in the kidneys and lungs and a parasitological study were conducted at different times of infection. Whatever the plasmodial species, the filarial recovery rate was strongly decreased. The peak of parasitaemia in the plasmodial infection was decreased in the course of *P. yoelii* infection but not in that of *P. chabaudi*. Regarding pathological lesions, *L. sigmodontis* can reverse lesions in the kidneys due to the presence of both *Plasmodium* species but does not modify the course of pulmonary lesions. The filarial infection induces granulomas in the lungs.

## Abstract (fr)

Les infections par de multiples parasites d’espèces différentes sont une situation normale plutôt qu’exceptionnelle chez les animaux comme chez l’homme. Les infections par des filaires et *Plasmodium* spp. sont des sujets importants en santé publique et sont souvent co-endémiques. Les relations entre ces parasites sont complexes. Les mécanismes reliant la modulation de la mise en place de l’infection plasmodiale ainsi que le succès de l’infection filarienne sont très peu connus. Malgré une activité croissante ces dernières années, les études comparant les co- et les mono-infections en sont à leur début et les résultats sont à première vue contradictoires. Dans cette étude sur des souris BALB/c, nous avons réalisé des infestations contrôlées et simultanées par la filaire *Litomosoides sigmodontis* et par *Plasmodium* spp. (*Plasmodium yoelii* 17XNL *ou Plasmodium chabaudi* 864VD). Des analyses anatomopathologiques dans les reins et les poumons ainsi qu’une étude parasitologique ont été menées à différents moments de l’infection. Le rendement filarien a été fortement diminué indépendamment de l’espèce plasmodiale co-infectante. Le pic de parasitémie de l’infection plasmodiale est diminué chez les souris infectées par *Plasmodium yoelii* mais pas chez les souris infectées par *Plasmodium chabaudi*. Quant aux lésions pathologiques, *L. sigmodontis* peut réverser les lésions rénales due à la présence du *Plasmodium* mais ne modifie pas celle observées dans les poumons. L’infection filarienne induit la formation de granulomes dans les poumons.

## Introduction

The prevalence of helminth infections is high in areas of malarial infections. Many cases of co-infection have been described, some with conflicting findings. In some cases, there is a reduction of the pathogenicity associated with malaria [7, 37], while in other cases there is an exacerbation of the disease [29, 38] or an increased prevalence of the *Plasmodium* [9, 47].

Tissue destruction is a common manifestation of many helminth infections and malarial infections, thus limiting parasite-mediated damage is critically important in diminishing disease sequelae. An inappropriate immune response can cause tissue pathologies by, amongst other things, inflammation: for example, many cases of malarial infection have been reported as causing acute renal failure and glomerulonephritis [15, 17, 24, 45], and chronic kidney damage [19, 49, 57], as well as acute lung injury and acute respiratory distress syndrome [52, 54]. Lung and kidney lesions have also been determined in mice infected with a lethal versus a non-lethal strain of *P. yoelii* (Landau & Killick-Kendrick, 1966) [27] in BALB/c mice [14]. Regarding helminth infections, both *Ascaris* and *Nippostrongylus* can lead to damage of the lung tissue during migration through the host. Interestingly, well-described Th2 responses against helminthic parasites [32] can result in tissue repair. The Th2 cytokines, IL-4 and IL-13, for example, are potent inducers of molecules involved in wound-healing processes, such as resistin-like-molecule-*α* (RELM*α*), arginase and matrix metallopeptidase 12 (MMP12) [39]. One recent proposal is that Th2 responses may have emerged as a tissue repair mechanism rather than being primarily anti-parasitic [3].

Filarial helminths can induce the production of anti-inflammatory cytokines such as IL-10 [11, 31] or TGF-*β* [11]. Filariae promote the secretion of IL-10 by CD25^hi^Foxp3^+^ T cells [18, 35], which results in a downregulation of the secretory pathway of IL-12p70/INF-*γ*, leading to a decrease in IFN-*γ*; TNF-*α* is also lowered [35]. Both INF-*γ* and TNF-*α* play an essential role in the resistance to *Plasmodium falciparum* (Laveran, 1880) [28]: IFN-*γ* mediates specific immunity to malaria [34, 35] and TNF-*α* is involved in the rapid clearance of *Plasmodium* [18, 23].

The microfilarial patent phase in the murine filarial model *Litomosoides sigmodontis* (Chandler, 1931) [8, 40] has opposite consequences on the outcome of *P. berghei* (Vincke & Lips, 1948) [56] and *P. chabaudi* (Landau, 1965) [25] infection in mice. Firstly, an improvement in the pathology of *P. berghei* through the production of IL-10 [13, 46] was observed in BALB/c mice [13] and in C57BL/6 mice [46]. In contrast, an exacerbation of parasitaemia, anaemia and weight loss in mice was observed in *P. chabaudi* infection in BALB/c mice [16]. This exacerbation was more pronounced in amicrofilaremic mice [16].

Although there are a few studies analysing the consequences of the filarial patent phase on plasmodial infection [13, 16, 46], none has studied the consequences of simultaneous co-infections on each parasite’s survival/development and tissue damage, i.e., in a context of migration of infective larvae inducing a Th2-driven response. We used the *L. sigmodontis* murine model co-infected by a non-lethal strain of *Plasmodium*, either one infecting preferentially reticulocytes (*P. yoelii*), or one infecting preferentially mature red blood cells (*P. chabaudi*). We compared the pathological states of lungs and kidneys of mice infected by *L. sigmodontis*, *P. chabaudi* or *P. yoelii* with the mice co-infected with *L. sigmodontis* and either *P. chabaudi* or *P. yoelii*.

## Materials and methods

### Ethics statement

All animal experiments were carried out in accordance with the EU Directive 2010/63/UE and the relevant national legislation, namely the French “Décret no 2013-118, 1^er^ février 2013, Ministère de l’Agriculture, de l’Agroalimentaire et de la Forêt”.

National licence number 75-1415 approved animal experiments: protocols were approved by the ethical committee of the Museum National d’Histoire Naturelle (Cometh Cuvier, Licence: 68-002) and by the “Direction départementale de la cohésion sociale et de la protection des populations” (DDCSPP) (No. 75-05-15).

### Parasites, mice, infections

Cryopreserved blood containing *P. yoelii yoelii* 17XNL clone 1.1 or *P. chabaudi chabaudi* 864VD with 5% glycerol was defrosted and used to inoculate ICR-CD1 mice, bred in the MNHN animal facilities. Retro-orbital terminal exsanguination was performed at the peak of parasitaemia. Aliquots were adjusted to 10^7^ parasitised red blood cells (pRBC) per mL, in a modified Alsever’s solution (dextrose: 20.5 g; trisodium citrate dihydrate: 7.9 g; NaCl: 4.2 g; glycerol: 100 mL; H_2_O: 900 mL/pH = 6.1). The aliquots were frozen at −80 °C.

The filariae *L. sigmodontis* were maintained in the MNHN laboratory and infective third-stage larvae (L3) were recovered by dissection of the mite vector *Ornithonyssus bacoti* (Hirst 1913) as previously described [10, 40].

Six-week-old female BALB/c mice were purchased from Harlan (France) and maintained in the MNHN animal facilities. Mice were divided into six groups. Group 1 comprised uninfected mice. Group 2 mice were inoculated with 40 L3 in 200 μL of RPMI 1640 subcutaneously into the left lumbar area of each mouse. Group 3 and 4 mice were injected intravenously with 10^6^ red blood cells infected with either *P. yoelii* or *P. chabaudi*. Group 5 and 6 mice were concomitantly infected with both *L. sigmodontis* and either *P. yoelii* or *P. chabaudi*.

The kinetics of infections was followed over 30 days. Mice were sacrificed at day 4 (first L3 localised to the pleural cavity), day 7 (the filarial recovery rate is maximum), day 13 (peak of parasitaemia for plasmodial infection) and day 30 (last moult and peak of cellular pleural infiltrate in filarial infection) post-inoculation (p.i.).

### Filarial load and plasmodial parasitaemia

The mice were anaesthetised and sacrificed by terminal bleeding. The pleural cavity was washed with 10 mL of cold phosphate-buffered saline (PBS), as previously described [33]. The following filarial features were analysed by light microscopy on filariae fixed *in toto* with 4% formaldehyde in cold PBS to avoid body shrinkage: (i) L4/moult 4/adults; (ii) gender.

Blood smears were obtained from the tail vein every two days, fixed in methanol, stained with Giemsa, and the percentage of *Plasmodium-*parasitised red blood cells was determined by optical microscopy (1000 red blood cells counted per blood smear).

### Histology and immunohistology

Mice from the six groups were necropsied on days 4 and 7 to investigate the migratory pulmonary phase of the filariae and on days 13 and 30 to visualise lesions in the kidney and the lungs.

At day 4 and day 7 mice were firstly exsanguinated then the lungs were expanded with RCl2^®^-CS100 (Alphelys, Plaisir, France) diluted in ethanol according to the manufacturer’s instructions. Lungs were removed and fixed for 24 h in this fixative.

At day 13 and day 30 mice were fixed *in toto* in Carnoy’s solution for 24 h to keep the parasitised red blood cells and the haemozoin-loaded white blood cells. Lungs and kidneys were removed and fixed for 24 h in this fixative.

In all cases, fixative was changed at 24 h post-fixation for a further 24 h. Thereafter, kidneys and lungs were removed from the fixative and placed in 70% alcohol for 2–7 days before paraffin embedding. Five-micron-thick serial sections were prepared. Sections of each tissue were then stained by Giemsa (Merck, Darmstadt, Germany)-colophonium (Wolbach 1911; Bray & Garnham 1962), Hemalun Eosin or Hemalun Eosin Safran (RAL, France).

CD3 and F4/80 immunostainings were performed on paraffin sections. T-cell staining was performed using the primary antibody against CD3 (rabbit polyclonal Ab, clone a0452, Dako France) at 1/100 dilution and a Leica Bond-max automat (dewaxing, antigen retrieval at pH9, Bond epitope retrieval solution 2, Leica) incubation and detection using Bond polymer Refine detection (which includes peroxide blocking, incubation by primary antibody for 20 min, post-primary, polymer, revelation by 3,3′-Diaminobenzidine (DAB) and counterstaining by haematoxylin). Macrophages were stained with the primary antibody against F4/80 (rabbit monoclonal Ab, clone BM8, Hycult Biotech) at 1/50 dilution. Antigen retrieval was performed at pH 6 (Antigen unmasking solution, Vector, France). Successive blockade of the tissue’s peroxidase (dual endogenous enzyme block, Dako, France) and biotin/avidin (avidin/biotin blocking kit, Vector, France) were realised before staining. Detection was then performed using the Vectastain Elite ABC kit (which includes a blocking serum for non-specific antigen, the biotinylated secondary antibody and the peroxidase). Revelation with AEC substrate and a quick counterstaining with Mayer’s haematoxylin were realised.

### Elisa

Sera (dilution 1:5) collected from individual mice were assayed for cytokine content by enzyme-linked immunosorbent assay (ELISA) in duplicate. These assays were performed according to the manufacturer’s recommendations, using INF-*γ*, TNF-*α* and the IL-10 ELISA kit (all from eBioscience SAS, Paris, France). Results are expressed as pg/mL. Detection limits were 15, 8 and 30 pg/mL for INF-*γ*, TNF-*α* and IL-10, respectively.

### Statistical analysis

The choice of statistical tests was based on sample size and on Bartlett’s test when normal distributions of the errors were expected. Data from separate experiments were pooled when possible. Results were analysed by *t*-test, one-way ANOVA or Kruskall-Wallis test in order to determine the effect of 1 factor, i.e., the group of mice, or two-way ANOVA in order to determine the effects of two factors, i.e., the group of mice and the time. Bonferroni’s or Dunn’s multiple comparisons post-tests were used to compare mono-infected groups with co-infected groups. Representation and data analyses were performed with the GraphPad Prism 5 software. Statistically significant values are indicated as follows: *p < 0.05; **p < 0.01; and ***p < 0.001.

## Results

### Co-infection restrains filarial load and plasmodial *P. yoelii* parasitaemia

**Figure 1.**
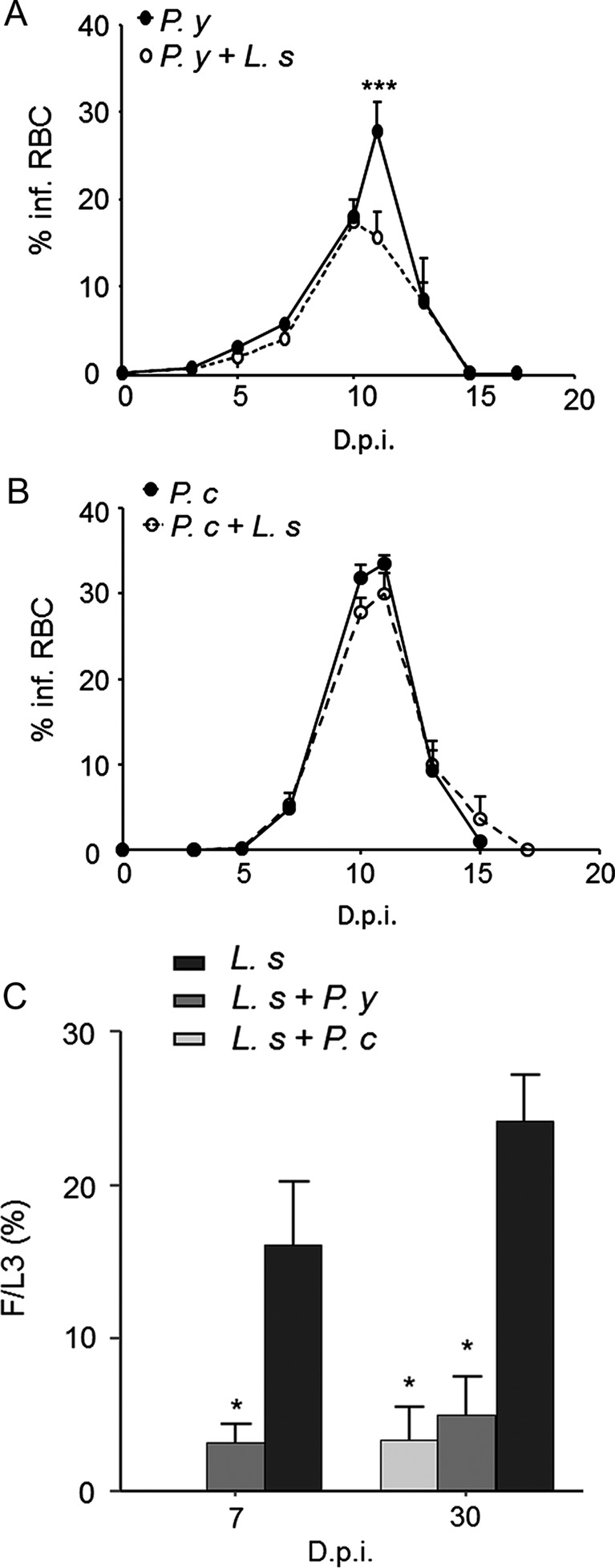
Parasitological monitoring. (A) Parasitaemia of the *P. yoelii* (*P. y*) infection in BALB/c mice. Five independent experiments pooled together, carried out with 4–7 mice per group. (B) Parasitaemia of the *P. chabaudi* (*P. c*) infection, *n* = 6 mice per group. % infRBC: Percentage of infected red blood cells; D.p.i.: Days post-inoculation; results are expressed as mean ± SEM; the differences between the mono-infected and co-infected mice with *L. sigmodontis* (*L. s*), and the modifications during the time course of the infection were analysed by a two-way analysis of variance. For each analysis the factor group and time effects were significant. The comparison among the groups for each time point was further assessed by Bonferroni’s multiple comparison test. * represents significant differences between the mice infected by *P. y* and by *P. y* + *L. s* (***p < 0.001). (C) Filariae were recovered in the pleural cavity at 7 and 30 days post-inoculation in BALB/c mice; F/L3: recovery rate of filariae, expressed as 100 × number of worms recovered/number of larvae inoculated; D.p.i.: Days post-inoculation; results are expressed as mean ± SEM; *n* = 6–8; Comparison between the groups of mice infected by *L. s* only or co-infected with *P. y*, at day 7, was assessed by a t test (*p < 0.05); at day 30, a one-way analysis of variance revealed a difference between the group of mice infected by *L. s* only and the mice co-infected both with *P. y* and *P. c* (*p < 0.05). The comparison was further assessed by Bonferroni’s test.

Filariae were recovered from the pleural cavity 7 and 30 days (D7 and D30) post-infection. As previously described, the *L. sigmodontis* (*L. s*) recovery rate in BALB/c mice was similar at both time points and was approximately 20% (16% ± 3.2 at D7, 24% ± 3.0 at D30). In mice co-infected with *P. yoelii* (*P. y*), the recovery rate decreased significantly at both D7 and D30 to 3.1% ± 1.2 and 5% ± 2.5, respectively (Fig. 1C). Similarly, in mice co-infected with *P. chabaudi* (*P. c*), the recovery rate dropped at D30 to 3.3% ± 2.2.

For both *Plasmodium* species, the course of infection occurred as follows: from day 1 to day 4, the parasitaemia increased slowly, then faster up to the peak of parasitaemia. The crisis occurred from day 9 to day 12. Finally, the parasitaemia decreased to become undetectable from day 17 to day 30. No recrudescence occurred (Figs. 1A and 1B) during these 13 days. The peaks of parasitaemia of *Plasmodium* reached 22.5% ± 1.1 and 33.5% ± 3.6 in mice infected by *P. y* and *P. c*, respectively. The peaks of parasitaemia of the mice co-infected with *L. s* were 16.2% ± 0.9 and 30% ± 3.2 with *P. y* and *P. c*, respectively.

### Lung anatomopathological analysis reveals a decrease in filarial granuloma in co-infected mice

**Figure 2.**
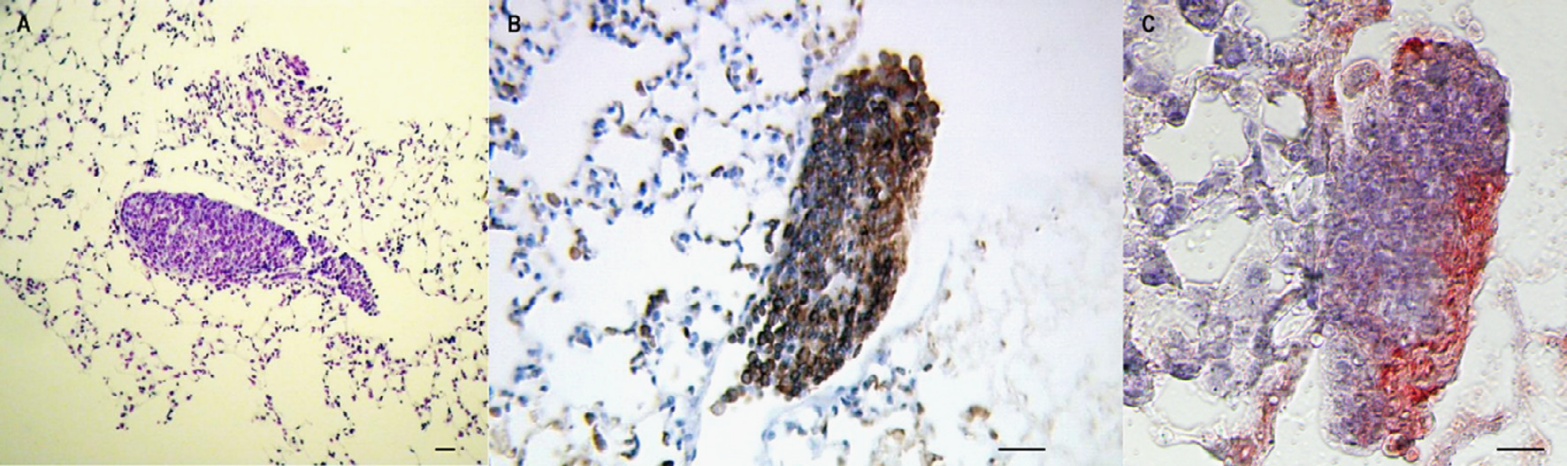
Granulomas in *L. sigmodontis*-infected mice are only detectable at 7 days post-infection. (A) Giemsa-colophonium staining of paraffin-embedded granulomas in the lungs of *L. sigmodontis* (*L. s*)- or *P. yoelii* (*P. y*) + *L. s*-infected BALB/c mice. (B) Immunohistochemical staining with antibody anti-CD3 of paraffin-embedded granulomas in the lung of infected BALB/c with *L. s* or *P. y* + *L. s*, revealed by HRP-DAB (counterstained with haematoxylin). (C) Immunohistochemical staining with antibody anti-F4/80 of paraffin-embedded granulomas in the lung of infected BALB/c with *L. s* or *P. y* + *L. s*, revealed by AEC counterstained with haematoxylin (Differential interference contrast (DIC) microscopy). Scale bars represent 50 μm.

**Figure 3.**
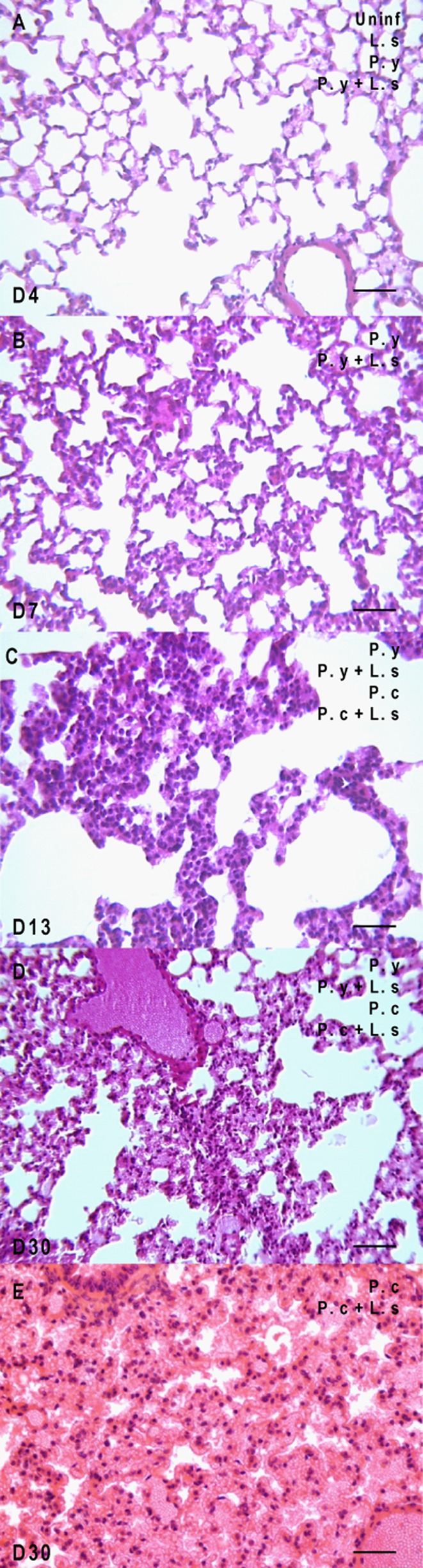
The lung injuries are progressive during the course of infection. Uninfected mice (uninf.), mice infected by *L. sigmodontis* (*L. s*), mice infected by *P. yoelii* (*P. y*) and mice co-infected by *P. y* + *L. s* were analysed throughout the infection from day 4 (D4) to day 30 (D30) post-infection; mice infected by *P. chabaudi* (*P. c*) and mice co-infected by *P. c* + *L. s* were analysed at the later time points, e.g., D13 and D30. (A) Normal lung at day 4 in all groups. (B) Lung with an increased cellular density at day 7 (D7) in *P. y*-infected mice, with or without *L. s*. (C) Lung with congestion and high number of cells at day 13 (D13) in all plasmodial infected groups. (D) Lung with higher congestion and high number of cells (D30) in all plasmodial infected groups. E. Lung with heavy congestion and haemorrhagic alveolitis at day 30 (D30) in *P. c*-infected mice, with or without *L. s*. Scale bars represent 50 μm.

No lesions were observed in either uninfected mice or in *L. s*-infected mice (Fig. 3A), except for the presence of intrapulmonary extravascular granulomas (Fig. 2A) mainly composed of T cells (Fig. 2B) and macrophages (Fig. 2C). These granulomas were observed in about 70% of *L. s*-infected mice instead of 28% in *P. y* + *L. s* co-infected mice only at D7 (Table 1). They were seen neither in uninfected mice nor in mice monoinfected with *Plasmodium* at any time point. An increase in the density of alveolar cells was also observed in the lungs of all mice infected with *P. y* + *L. s* and *P. y* (Fig. 3B) at D7. At D13 and D30, the density of alveolar cells was increased in all malaria-infected mice, co-infected with *L. s* or not.

**Table 1.** Detailed chronological histopathological results in the lungs.

		Lesions			
Groups	Time (D.p.i.)	Leucocytes infiltrates	Cells increased	Congestion	Haemorragic alveolitis
*N*	4	0	0	0	0
	7	0	0	0	0
	13	0	0	0	0
	30	0	0	0	0
*P. y*	4	0	0	0	0
	7	0	5/5	0	0
	13	0	3/3	3/3	0
	30	0	3/3	3/3	0
*P. c*	13	0	3/3	3/3	0
	30	0	4/4	4/4	4/4
*P. y* + *L. s*	4	0	0	0	0
	7	2/7	7/7	0	0
	13	0	3/3	3/3	0
	30	0	2/2	2/2	0
*P. c* + *L. s*	13	0	2/2	2/2	0
	30	0	3/3	3/3	3/3
*L. s*	4	0	0	0	0
	7	5/7	0	0	0
	13	0	0	0	0
	30	0	0	0	0

The different groups of mice are listed in column 1. Time of necropsies is indicated in the second column. For each lesion observed in the lungs, numbers indicate the number of mice showing the lesions/total number of studied mice. D.p.i.: Day post-inoculation; Alv cells inc: increase in the number of alveolar cells; uninf: uninfected mice; *L. s*: mice infected by *L. sigmodontis*; *P. y*: mice infected by *P. yoelii*; *P. y* + *L. s*: mice co-infected by *L. sigmodontis* and *P. yoelii*; *P. c*: mice infected by *P. chabaudi*; *P. c* + *L. s*: mice co-infected by *L. sigmodontis* and *P. chabaudi*.

At D13, the lungs were congested in all malaria-infected mice, whether co-infected with *L. s* or not (Fig. 3C). However, at D30, the lungs of mice infected with *P. y* and *P. y* + *L. s* were highly congested (Fig. 3D), while those of mice infected with *P. c* and *P. c* + *L. s* were both congested but also showed haemorrhagic alveolitis (Fig. 3E).

### Kidney anatomopathological analysis reveals a decrease in malaria-associated glomerular pathologies in co-infected mice

**Figure 4.**
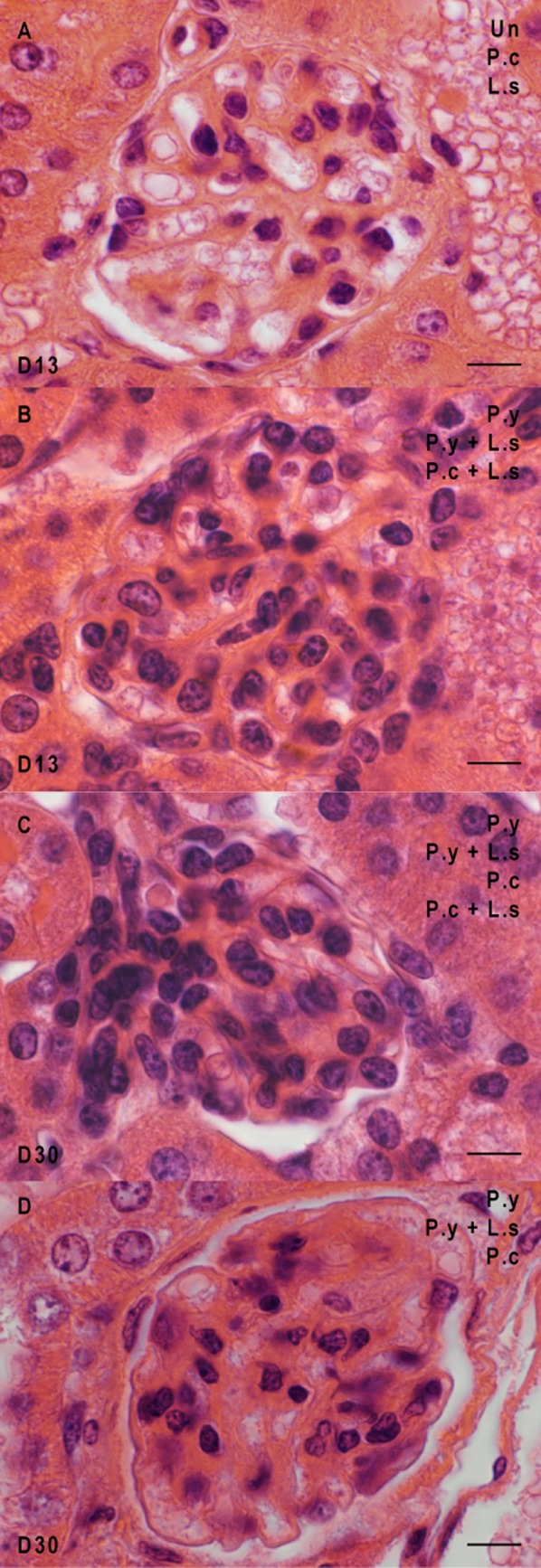
Glomerulonephritis in the kidneys of the mice at different times of infection. Glomeruli from uninfected mice (uninf.), mice infected by *L. sigmodontis* (*L. s*), mice infected by *P. yoelii* (*P. y*), mice infected by *P. chabaudi* (*P. c*), mice co-infected by *P. y* + *L. s* and mice co-infected by *P. c* + *L. s* were analysed at two time points, e.g., D13 and D30. (A) Normal glomeruli at day 13 post-infection. (B) Proliferation of the mesangial cells at day 13 in both co-infected groups and in *P. y*-infected mice. (C) Proliferation of the mesangial cells at day 30 in both plasmodial infected groups and in both co-infected groups. (D) Obliteration of the capillaries at day 30 in both plasmodial infected groups and in the mice co-infected with *L. s* and *P. y*. Scale bars represent 10 μm.

The proliferation of the mesangial cells is the most frequently observed phenomenon in the glomeruli (Figs. 4B and 4C). At day 13, 20% of the glomeruli of *P. y*-infected mice and 12% of *P. y* + *L. s* co-infected mice harboured this type of lesion. It was not observed in any other group of mice. Yet by day 30, 18% of the glomeruli of *P. y*-infected mice showed that lesion while only 2% in the *P. y* + *L. s* group carried it. This lesion also appeared in 20% of *P. c*-infected mice but only in 5% of *P. c* + *L. s* co-infected mice (Table 2).

**Table 2.** Detailed chronological histopathological results in the glomeruli.

		Phenomenon observed		
Groups	Time (D.p.i.)	Obliterated capillaries	Mesangial cells proliferation	Macrophages with pigment
*N*	13	0	0	0
	30	0	0	0
*P. y*	13	3/3	3/3	3/3
		14	20	72
	30	0	3/3	0
			18	
*P. c*	13	0	0	0
	30	3/6	6/6	0
		12	20	
*P.y* + *L. s*	13	1/3	3/3	3/3
		9	12	54
	30	0	1/3	0
			6	
*P. c* + *L. s*	13	0	1/3	0
			9	
	30	0	1/2	0
			10	
*L. s*	13	0	0	0
	30	0	0	0

The different groups of mice are listed in column 1. Time of necropsies is indicated in the second column. For both types of lesions observed in the kidneys, the ratio of mice with lesions to the total number of mice studied is indicated, as well as the mean number of glomeruli showing the lesions. D.p.i.: Day post-inoculation; prolif. proliferation; uninf: uninfected mice; *L. s*: mice infected by *L. sigmodontis*; *P. y*: mice infected by *P. yoelii*; *P. y* + *L. s*: mice co-infected by *L. sigmodontis* and *P. yoelii*; *P. c*: mice infected by *P. chabaudi*; *P. c* + *L. s*: mice co-infected by *L. sigmodontis* and *P. chabaudi*.

As a consequence, obliteration of the capillaries was observed in the glomeruli. At day 13, it was observed in 100% of glomeruli of *P. y*-infected mice, in 33% of *P. y* + *L. s* co-infected mice, and not in any other groups. At day 30, only 50% of *P. c*-infected mice were found to have that type of lesion.

### Kidney macrophages and blood monocytes from both *P. yoelii*-infected and co-infected mice contain haemozoin pigment

**Figure 5.**
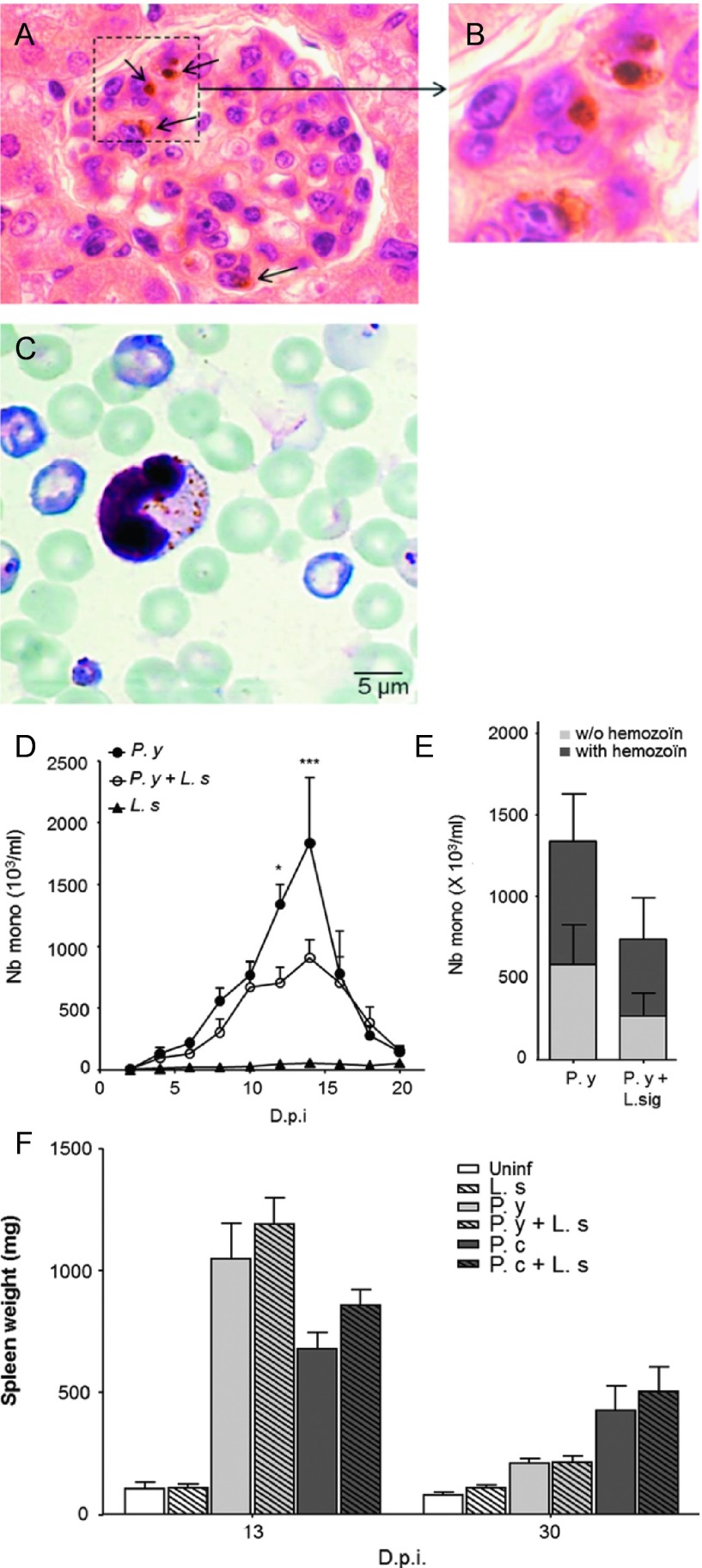
Phagocytosed haemozoin pigment in tissular macrophages and circulating monocytes. (A) Sections of glomeruli observed at day 13 in the kidneys of mice infected with *P. yoelii* (*P. y*) or co-infected with *P. yoelii* and *L. sigmodontis* (*P. y* + *L. s*), showing macrophages containing phagocytosed plasmodial haemozoin pigment (arrows). (B) Zoom magnification of a macrophage with haemozoin pigment. (C) Monocytes with haemozoin pigment in the blood of mice infected with *P. y* or *P. y* + *L. s*, at day 13. (D) Kinetics of the circulating monocytes in the blood of infected mice; *n* = 10; the differences between the mono-infected and co-infected mice, and the modifications during the time course of the infection were analysed by a two-way analysis of variance. For each analysis the factor group and time effects were significant. Comparison between the groups for each time point was further assessed by Bonferroni’s multiple comparison test. Significant differences between the mice infected by *P. y* and by *P. y* + *L. s* are reported (*p < 0.05; ***p < 0.001). (E) Enumeration of the monocytes at the peak of the kinetics, containing haemozoin or not. Results are expressed as mean ± SEM; *n* = 10; the differences between the mono-infected and co-infected mice, and between the monocytes containing haemozoin and the monocytes containing no haemozoin were analysed by a two-way analysis of variance. No statistical significance was observed. Comparison between the groups was further assessed by Bonferroni’s multiple comparison test (*p* > 0.05). (F) Splenomegaly in mice infected with *P. c* and *P. y*. Results are the mean of spleen weight ± SEM at 13 and 30 days post-inoculation (D.p.i.), (*n* = 3–4).

Macrophages in glomeruli were observed to contain haemozoin pigment only in *P. y*-infected mice and in *P. y* + *L. s* co-infected mice (Figs. 5A and 5B).

Circulating leukocytes including monocytes increased during plasmodial infections. The highest number of monocytes in the blood was observed between the 13th and the 16th days after infection. This peak was significantly lower in the *P. y* + *L. s* co-infected mice (907 × 10^3^ ± 144 × 10^3^/mL) than in *P. y*-infected mice (1835 × 10^3^ ± 526 × 10^3^/mL). No increase was observed in *L. s*-infected mice (Fig. 5D).

Monocytes containing phagocytosed erythrocytes parasitised by *Plasmodium* were identified by the parasite pigment haemozoin in both *P. y*-infected and *P. y* + *L. s* co-infected mice (Fig. 5C) but not in *P. c*-infected mice. Monocytes with haemozoin were more numerous in *Plasmodium* mono-infected mice than in co-infected mice around the peak of parasitaemia at D11 (*p* < 0.05) and D13 p.i. (*p* < 0.001: 582 × 10^3^ ± 77 × 10^3^/mL compared with 269 × 10^3^ ± 43 × 10^3^/mL) (Fig. 5E).

### Splenomegaly in plasmodium-infected mice

Splenomegaly was observed at D13 p.i and D30 p.i. in all the groups of mice infected with *Plasmodium* sp. At D13 p. i. a 10-fold increase and a 7-fold increase were demonstrated in *P. y*-infected mice and *P. c*-infected mice, respectively. The co-infection with *L. s* did not modify these levels of splenomegaly (Fig. 5F). At D30 p.i. a 2-fold increase and a 4-fold increase were still noticeable in *P. y*-infected mice and *P. c*-infected mice, respectively. The co-infection with *L. s* did not modify these levels of splenomegaly (Fig. 5F).

### Pro-inflammatory seric cytokines are increased in co-infected mice compared with filarial-infected mice at day 7, but not IL-10

**Figure 6.**
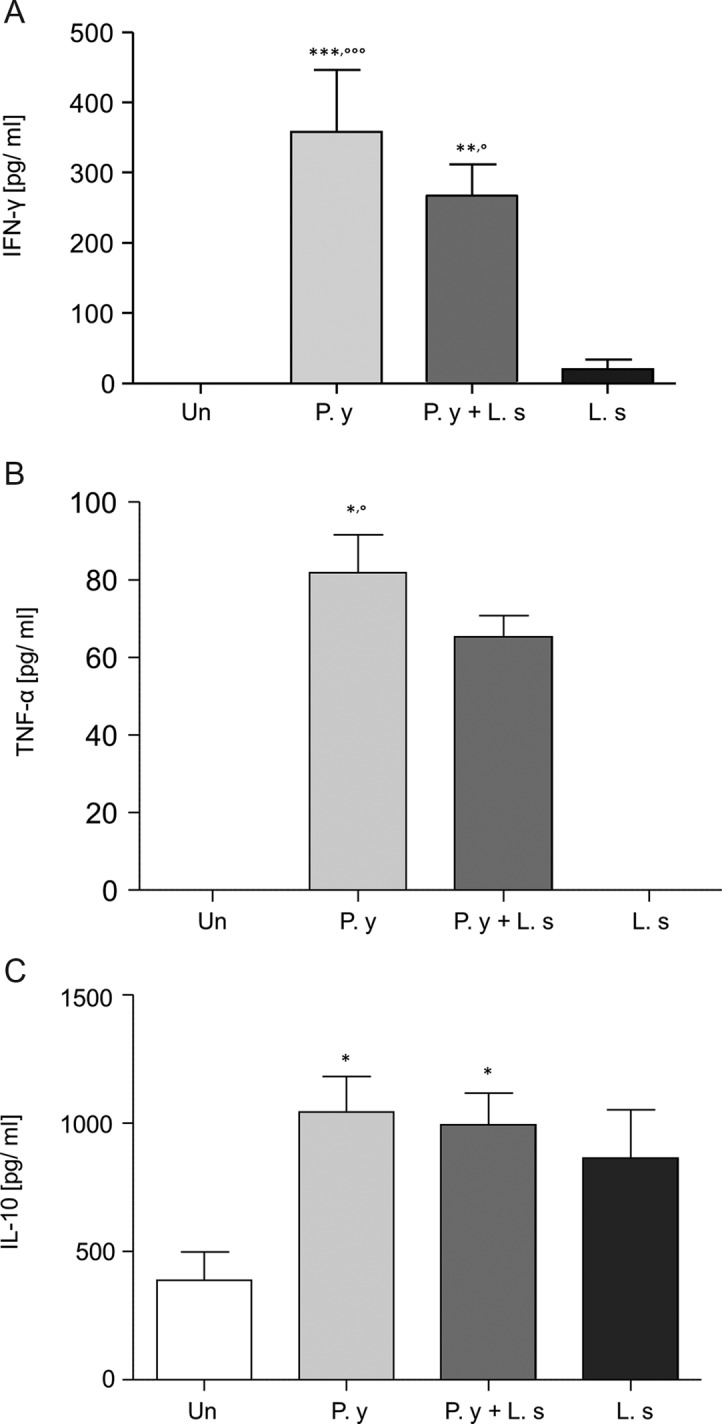
Seric proinflammatory cytokines IFN-*γ* and TNF-*α* are produced in co-infected or in *P. y*oelii-infected mice. Measurement of cytokines in the sera of the different group of BALB/c mice at day 7 after inoculation of either 40 L3 of *L. sigmodontis* or 10^6^ iRBC by *P. yoelii* or both or uninfested. (A) Level of IFN-*γ*. Two independent experiments pooled together, carried out with four mice per group per experiment. A one-way analysis of variance revealed a difference between the groups of mice infected by *P. y*. or co-infected with either the uninfected mice (*) or the mice infected with *L. s* (°). The comparisons were further assessed by Bonferroni’s multiple comparison test (°*p* < 0.05; **p < 0.01; ***^,^°°°*p* < 0.001). (B) Level of TNF-*α*. *n* = 4 mice per group. A Kruskall-Wallis test revealed a difference among the groups of mice infected by *P. y* and either the uninfected mice (*) or the mice infected with *L. s* (°). The comparison was further assessed by Dunn’s multiple comparison test (*^,^°*p* < 0.05) (C) Level of IL-10. Two independent experiments pooled together, carried out with four mice per group per experiment. A one-way analysis of variance revealed a difference between *P. y*-infected mice and the uninfected mice. The comparison was further assessed by Bonferroni’s multiple comparison test (*p < 0.05). Results are the means of measurements ± SEM.

Seric concentrations of IFN-*γ* and TNF-*α*, two pro-inflammatory cytokines, and IL-10, an anti-inflammatory cytokine, were monitored at day 7 in the different groups of mice, the earliest time point showing a lower filarial recovery rate in co-infected mice than in filarial-infected mice. IFN-*γ* and TNF-*α* were not detected in the naïve mice or in the mice infected with *L. sigmodontis* (Figs. 6A and 6B). In the presence of *Plasmodium*, circulating levels of IFN-*γ* and TNF-*α* are increased in both *P. y* and in *P. y* + *L. s* (Figs. 6A and 6B). Levels of the IL-10 measured in the infected groups were similar (Fig. 6C).

## Discussion

The data presented in this study show that simultaneous infection of *Litomosoides sigmodontis* with *P. yoelii* or *P. chabaudi* restrains filarial load in BALB/c mice. In these co-infected mice, the decrease in malarial parasitemia is strain-dependent: only the peak of parasitaemia with *P. yoelii*, but not that of *P. chabaudi*, was lowered. The extent of some tissue damage also depends on the infectious status. Glomerular pathologies due to *Plasmodium* are malaria strain-independent, but they are less frequent when the mice are co-infected with filariae. The presence of haemozoin pigments in monocyte and renal macrophages is malarial strain-dependent; they were only observed in mice infected with *P. y*. Lung granulomas were repeatedly observed only in filariae-infected mice and rarely in co-infected mice. However, other lung damage increased over the course of infection, whatever the strain of malaria and with or without filariae.

The outcome of filaria/Plasmodium co-infection is strongly malaria strain-dependent but the strain of mice or the time between the two infections (i.e., simultaneous or consecutive infection) are less critical. For example, the outcome of *P. berghei* (*P. b*) infection is similar in BALB/c and C57BL/6 mice [13, 46]. In addition, in both BALB/c [13] and C57BL/6 [46] mice the parasitaemia is lower in mice co-infected with *P. b* and *L. s*. Regarding the strain of malaria in co-infected mice, an increase in the parasitaemia was observed in BALB/c mice inoculated with *P. c* + *L. s* instead of a decrease with *P. b* + *L. s* [16]. Moreover, the parasitaemia is even stronger if the mice in the patent phase are amicrofilaremic [13, 16]. The above studies initiated the plasmodial infestation during the patent phase of *L. s*, whereas in our model, the co-infection was performed simultaneously, therefore in a different immune environment. The parasitaemia of *P. c* in our study was not altered, whereas in the study of Graham et al. it was exacerbated [16]. In addition, we used the 864VD strain, whereas Graham *et al*. used the clone “AS” from the 399BY strain [16] which is known for its higher virulence. We also observed that the parasitaemia of *P. y* was reduced in co-infected mice. This could be related to the results obtained by Fernandez-Ruiz et al. [13] with *P. b* because *P. y* and *P. b* are closely related rodent species both in their asynchrony [26] and in their intimate phylogenetic relationships as they belong to a monophyletic group [42].

The filarial survival in mice is also modified by the presence of a plasmodial infection. The number of filariae recovered from the pleural cavity at 30 days post-infection was reduced by 80% in the co-infected mice compared with filarial-infected mice. Infective larvae (L3) migrate from the skin to the pleural cavity [40] but only 20–25% of a 40 L3 inoculum, subcutaneously delivered, make their way to the pleural cavity of mice [40]. These successful L3 arrive in the pleural cavity by day 6–8 post-infection [4, 20]. The reduced filarial recovery rate in co-infected mice was observed as early as 7 days post-infection, suggesting a higher reduction of L3 in the skin or during their migratory phase to reach the pleural cavity.

One could argue that modifications of the immune environment could explain these changes in the parasites’ outcomes. The ability of *L. s* to induce Th2-type immune responses is well documented [1, 21]. Although Th2 responses seem capable of mediating the destruction of the larval stages at the site of delivery [33], both Th1 and Th2 may be needed to contain the adult stage [1, 2, 32]. A regulatory response is also mounted in filarial infection [43, 44, 50, 51], reducing the production of IFN-*γ* and TNF-*α* [34]. During a plasmodial infection, IFN-*γ* and TNF-*α* [41, 55] are produced and Th1 cells [59] are involved in immunity against blood stages. In our study, the levels of these two inflammatory cytokines are higher than in the mice infected only with *L. s.* The presence of *Plasmodium* could modify the production of IFN-*γ* and TNF-*α* and this phenomenon could result in the reduction in the number of filariae.

Pathology associated with all malarial species is related to the rupture of infected erythrocytes and the release of parasite material and metabolites, haemozoin (malaria pigment) and cellular debris. Tissue pathologies in malaria have been described in humans suffering from chronic or severe malaria; glomerulonephritis with *P. falciparum* or chronic nephrosis with *P. malariae* [5, 6], acute lung injury with damage to the cell membrane, and oedema describing the acute respiratory distress syndrome [36, 53].

Studies on ICR mice infected by *P. c* (strain 864 VD) or by *P. yoelii nigeriensis* (closely related to *P. yoelii yoelii*) have revealed many lesions, for example in the kidneys [57]. In agreement with our observations, lesions in the kidneys appeared, either after the peak of parasitaemia with *P. y* or late with *P. c* [57] in BALB/c mice and ICR mice [58], the latter being more susceptible to parasite infection as a higher parasitaemia and a delayed peak were observed. These lesions were observed up to 116 days in *P. c*-infected ICR mice and up to 77 days in *P. y*-infected ICR mice and were classified as irreversible [57]. In our study, the co-infection with *L. sigmodontis* reduced the lesions in the kidneys, suggesting that the presence of the filariae might induce an anti-inflammatory process, reversing the lesions observed in the kidney. The increase in mesangial cells in the kidneys is responsible for the obliteration of the capillaries [22], very likely because these cells release a large number of pro-inflammatory compounds which are capable of causing such a phenomenon [22].

Haemozoin (malaria pigment) was observed in monocytes and kidney macrophages of *P. y*-infected mice only, at the peak of parasitaemia when the number of monocytes also peaks in the blood. A difference in the amount of haemozoin was also observed late (6 and 9 months post-infection) in the spleen of ICR mice infected with *P. c* and *P. y* (10-fold higher in mice infected with *P. y*) and is partly due to the accumulation of haemozoin-laden macrophages [30]; at the peak of parasitaemia our observations also show a higher spleen weight in mice infected with *P. y*, which could be explained by the presence of haemozoin-laden macrophages (Fig. 5F). This splenomegaly has previously been observed at the peak of parasitaemia [48] and in both plasmodial infections it decreases and is even stronger in *P. y*-infected mice. Moreover, the presence of *L. s* seems to result in a slightly higher splenomegaly in both *P. y* and *P. c* co-infected mice as observed at day 7 in C57BL/6 mice infected with *L. s* and 2 months later with *P. berghei* [46].

Pulmonary pathologies were observed through the course of infection, from day 7 to day 30, in *P. c*, *P. y* and in co-infected mice, even when *Plasmodium* parasites were no longer discernable in the blood. Oedemas appeared at day 13 post-infection. The kinetics of pulmonary lesions due to *Plasmodium* was the same in mice regardless of the infectious status. Oedemas have been reported at day 6 post-infection in BALB/c mice inoculated with *P. yoelii* 17XL, a lethal strain [14], but not with *P. yoelii* 17XNL (non-lethal), which is consistent with our results and emphasises the role of 17XL and 17XNL as models of acute lung disease and chronic pulmonary disorder, respectively. The increased density of alveolar cells in lungs, i.e., alveolar macrophages and type I and type II alveolar epithelial cells, could be sequentially involved in the control of parasite infection. For example, type I cells are known to be severely damaged under pathological conditions, whereas type II cells have been shown to play a key role in the cellular adaptation in response to lung injury [12], contributing to repairing the damaged alveoli and preventing their collapse.

A new phenomenon was observed in the lungs of infected mice and was associated with the presence of the filariae. Areas of leucocyte infiltrates in the lungs appeared frequently at day 7 post-infection in mice infected with filariae alone and very seldom in mice co-infected with *P. y*. They had disappeared at day 13. The egress of L3 in the pleural cavity is currently a black box and we think that filarial larvae may pass through the lung and the mesothelium during their migration phase from the site of inoculation to the pleural cavity, inducing such an inflammatory response with cellular recruitment.

To conclude, this study highlights that a simultaneous *Plasmodium*/filaria co-infection differentially regulates the malarial parasitaemia in a malaria strain-dependent way, the filarial load in a malaria strain-independent way and some of the tissue damage compared with a single infection. Moreover, the presence of filariae has a protective effect on the lesions in the kidney but not in the lungs.
